# A Revised View on the Role of Surface AMPAR Mobility in Tuning Synaptic Transmission: Limitations, Tools, and Alternative Views

**DOI:** 10.3389/fnsyn.2018.00021

**Published:** 2018-07-20

**Authors:** Jary Y. Delgado, Paul R. Selvin

**Affiliations:** ^1^Department of Neurobiology, The University of Chicago, Chicago, IL, United States; ^2^Department of Physics, Biophysics, and the Center for the Physics of Living Cells, University of Illinois at Urbana-Champaign, Urbana, IL, United States

**Keywords:** AMPAR, diffusion, short-term plasticity, desensitization, high-resolution microscopy

## Abstract

Calcium dynamics in presynaptic terminals regulate the response dynamics of most central excitatory synapses. However, this dogma has been challenged by the hypothesis that mobility of the postsynaptic alpha-amino-3-hydroxy-5-methyl-4-isoxazolepropionic acid subtype glutamate receptors (AMPAR) plays a role in tuning fast excitatory synaptic transmission. In this review, we reevaluate the factors regulating postsynaptic AMPAR mobility, reassess the modeling parameters, analyze the experimental tools, and end by providing alternative ideas stemming from recent results. In particular, newer methods of labeling AMPARs with small fluorophores in live neurons, combined with super-resolution microscopy and sub-second dynamics, lends support to the idea that AMPARs are primarily within the synapse, are greatly constrained, and have much slower mobility than previously thought. We discuss new experiments which may be necessary to readdress the role of postsynaptic AMPAR mobility in tuning fast excitatory synaptic transmission.

## Introduction

**FIGURE 1:**
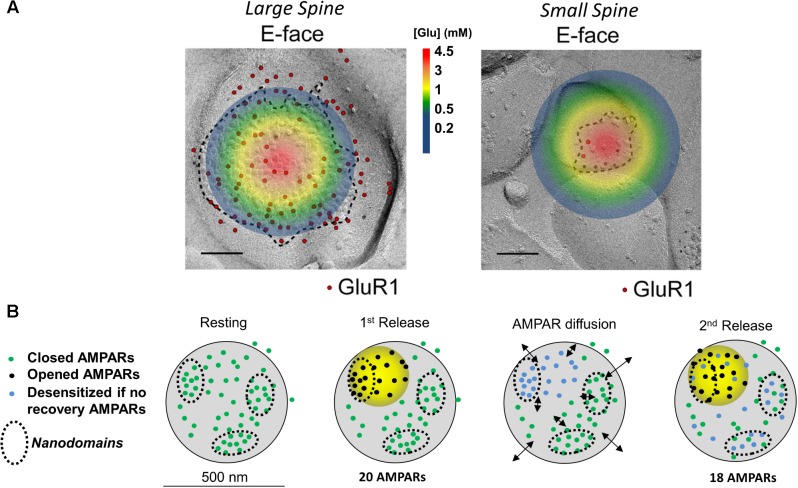
Image and legend reprinted from Shinohara and Hirase (2009). **(A)** Examples of paired SDS-digested freeze-fracture replica labeling (SDS-FRL) for GluR1 (E-face) at large vs. small spines in CA1 of stratum radiatum synapses (old nomenclature for AMPAR subunits is used). Colored circle represents the extent of glutamate spread 50 μs post vesicular fusion as predicted by Raghavachari and Lisman (2004). Approximate colored scaled for glutamate concentrations shown in between images (in mM). Scale bars: 100 nm. **(B)** Conceptual model of AMPAR mobility during paired-pulse stimulation. Diagram of a large mushroom spine with realistic AMPAR content randomly distributed thought the space in small nanodomains (black circles). Zone of high glutamate concentration is illustrated by the yellow circle and should follow the color schemed shown above. Three different states for AMPAR are assigned: closed (green color), opened (black), and desensitized (blue). Four states are shown: resting (left), 1st release, AMPAR diffusion, and 2nd release. Estimated synaptic response is shown above as a one to one correspondence between number of AMPAR and size of response.

Fast excitatory transmission at central nervous synapses depends on glutamate release from presynaptic terminals. Presynaptic glutamate release is under the control of Ca^2+^ channels which open after an incoming action potential invades the presynaptic terminal. The influx of Ca^2+^ then triggers the fusion of a primed glutamate-containing presynaptic vesicle. Results from modeling work indicate that once a vesicle fuses with the plasma membrane, glutamate concentrations quickly increase at the synaptic cleft, reaching an estimated 1-3 mM within 50 microseconds (Raghavachari and Lisman, 2004). The highest concentration of glutamate is at the site of vesicular fusion (illustrated in **Figure 1A**), where it then activates postsynaptically opposed glutamatergic receptors such as AMPARs. The activation of AMPARs is spatially restricted to a 125 nm radius from the center of glutamate release. This dynamic processes results in activation of only a fraction of all postsynaptic AMPARs (Tarusawa et al., 2009), wherein AMPARs positioned beyond the 125 nm radius don’t experience glutamate concentrations high enough to promote gating or desensitization (Trussell et al., 1993; Raghavachari and Lisman, 2004). However, an exception to this is at glomerular-type synapses, where glutamate can build up during trains of paired

stimulation and trigger postsynaptic AMPAR desensitization (Chen et al., 2002; DiGregorio et al., 2007; Budisantoso et al., 2012). At most excitatory synapses, the response dynamic to a pair of axonal stimuli is thought to solely depend on factors regulating presynaptic glutamate release, diffusion, and clearance. Whether the augmentation or reduction of excitatory synaptic transmission upon *paired-pulse facilitation* or *paired-pulse depression*, respectively, is not believed to involve postsynaptic factors (Zucker and Regehr, 2002; Christie et al., 2010; Regehr, 2012). Findings from Heine et al. (2008), Frischknecht et al. (2009), and Choquet (2010) have challenged this dogma by proposing a *post*synaptic component of the paired-pulse response. Their hypothesis supposes that postsynaptic AMPARs become desensitized in response to the first pulse of glutamate of the pair of axonal stimuli and that these desensitized AMPARs need then to be replaced by non-desensitized AMPARs via lateral diffusion (**Figure 1B**). The replacement of the desensitized postsynaptic AMPARs allows the synapse to respond to the second incoming stimulus and activate another pulse, generally somewhat smaller than the first, although occasionally equal or larger to it. Their hypothesis also supposes that the exchange of desensitized AMPARs via diffusion is faster than the rate of recovery of AMPARs from the desensitize state (Hestrin, 1992; Robert and Howe, 2003). The source of non-desensitized receptors was initially hypothesized to be at “extrasynaptic” membranes, however, this is no longer the favored hypothesis (Choquet, 2010; Choquet and Triller, 2013; Compans et al., 2016).

This revolution in thinking led by Choquet and colleagues about excitatory glutamatergic synapses has been possible with the advent of high-resolution single-particle microscopy techniques and electron microscopy (EM). These techniques have shown that surface-expressed AMPARs diffuse at synaptic and extrasynaptic areas of dendrites (Borgdorff and Choquet, 2002; Groc et al., 2004; Bats et al., 2007; Saglietti et al., 2007). The recent incorporation of super-resolution techniques and EM studies have shown that surface-expressed AMPARs lie in nanodomains, small clusters of about 70 nm in diameter (Nair et al., 2013; Cai et al., 2014; Constals et al., 2015; Li et al., 2016; Lee et al., 2017).

The nanodomain organization of synaptic AMPARs forms the basis for a newer model of how postsynaptic AMPAR mobility plays a role in recovering desensitized AMPARs during paired-pulse stimulation (Heine et al., 2008; Compans et al., 2016). In this model, the AMPAR nanodomains (1) determine the minimal unit of synaptic response, (2) are independent of each other, and (3) serve as a source of receptors that can supply non-desensitized AMPARs to the activated region (**Figure 1B**). At larger spines, AMPARs cluster into nanodomains which are served by a glutamate activation zone of about 250 nm in diameter (Raghavachari and Lisman, 2004; Heine et al., 2008; Compans et al., 2016). Because a single glutamate transient cannot reach all receptors on a large spine (Trussell et al., 1993), Compans and colleagues proposed that multiple zones of glutamate release are required to activate different nanodomains. Activated AMPARs within the nanodomain become desensitized (**Figure 1**, blue dots) and are then replaced by diffusion of naïve AMPARs (green dots) from adjacent nanodomains (**Figure 1**, black arrows). This idea of receptor exchange between intrasynaptic nanodomains is herein referred to as the nanodomain hypothesis. This challenged the longstanding belief that presynaptic mechanisms solely underlie the paired-pulse response.

In this review we will revisit the nanodomain hypothesis and the factors regulating postsynaptic AMPAR mobility and diffusion within nanodomains. We will particularly focus on understanding the dynamics of AMPAR exchange between intrasynaptic nanodomains and the role of AMPAR mobility in the paired-pulse synaptic response. We conclude that recent measurements, most notably super-resolution microscopy with small fluorescent labels, show that AMPARs do not have a enough freedom to move around the synapse, and hence, the nanodomain hypothesis, by itself, fails to explain the paired-pulse response. We present some other hypotheses which might help explain the observed changes in paired-pulse response.

## Biological Limitations of Ampar Mobility

### Spine Size

According to the nanodomain hypothesis, the role of AMPAR mobility in the paired-pulse response requires multiple AMPAR nanodomains to be present in the postsynaptic spine. Therefore, the size of the spine, which determines the number of nanodomains, should also determine how strongly it will be affected by glutamate (Shinohara and Hirase, 2009). (The number of AMPARs scales almost linearly with the size of the postsynaptic spine). If each spine is fully independent with no glutamate spillover occurring, the glutamate transients will more strongly impact smaller spines [postsynaptic densities (PSDs) < 0.05 μm^2^] than larger ones. Spines with small postsynaptic membranes (<0.05 μm^2^, diameters ∼250 nm or less) have a lower number of postsynaptic AMPARs than larger mushroom spines (Harris and Stevens, 1989; Arellano, 2007; Shinohara and Hirase, 2009; Fukazawa and Shigemoto, 2012). At small spines, as the one shown on **Figure 1** (area of ∼0.008 μm^2^), glutamate will quickly diffuse through the whole synaptic cleft and extrasynaptic membrane. This is expected to gate all postsynaptic AMPARs (**Figure 1**, top); a fraction of those will fully gate and others will desensitize without opening (Robert and Howe, 2003; Tarusawa et al., 2009). This leaves very few naïve AMPARs to replenish the spine, suggesting that at small synapses, which comprise ∼53% of all synapses, AMPAR mobility plays no role (Harris and Stevens, 1989; Arellano, 2007). Larger postsynaptic densities, which make up 47% of total synapses, could have independent nanodomains of AMPARs as seen in synapses with perforated PSDs (Harris et al., 1992). These independent nanodomains could, in principle, provide non-desensitized AMPARs to replenish the activated nanodomain (Choquet and Triller, 2013; Nair et al., 2013; Compans et al., 2016). Therefore, it seems that postsynaptic AMPAR mobility must be a phenomenon that can only work at larger spines with areas greater than 0.05 μm^2^ (Harris and Stevens, 1989).

### The PSD Is a Crowded Environment

**FIGURE 2:**
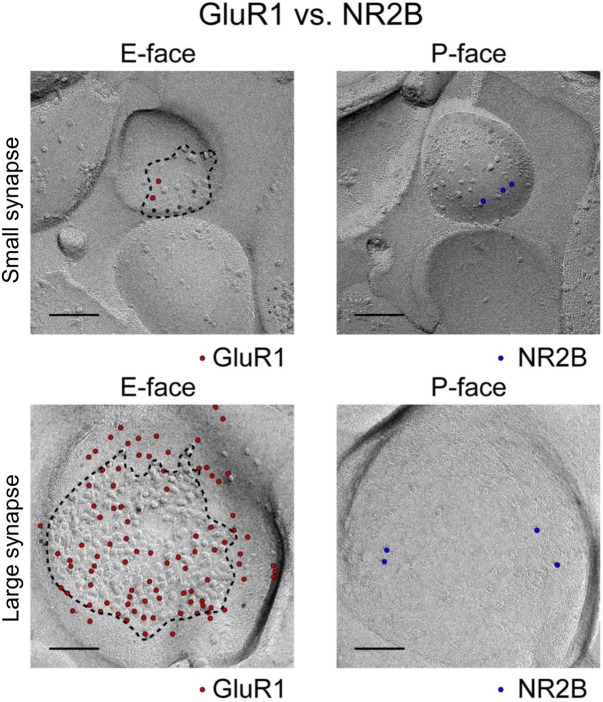
Image and legend reprinted from Shinohara and Hirase (2009). Examples of paired SDS-digested freeze-fracture replica labeling (SDS-FRL) for GluR1 (E-face) vs. NR2B (P-face) in CA1 stratum radiatum synapses (old nomenclature for glutamate receptors is used). Upper rows are examples of small synapses, whereas lower rows are that of large synapses. Boundaries of intramembrane particles (IMP) shown in dotted lines in E-faces correspond to exposed PSD areas (ePSD). Scale bars: 100 nm.

Intrasynaptic AMPAR diffusion during paired-pulse stimulation requires diffusion to happen very fast, with mean instantaneous diffusion (D_inst_) of 0.1 μm^2^/s (Heine et al., 2008; Freche et al., 2011; Czöndör et al., 2012). However, there are a number of factors that can limit the rate of diffusion within the synapse. In particular, the intracellular scaffolding proteins, of which there are many, slow down diffusion in two ways: by interacting with the auxiliary subunits associated with the AMPARs (TARPS) (Bats et al., 2007), and by creating barriers to free diffusion (Choquet and Triller, 2013; Li and Blanpied, 2016; Li et al., 2016). A given postsynaptic density contains a large number of intracellular scaffolding proteins. It is particularly rich in members of the membrane-associated guanylate kinase (MAGUK) family of scaffold proteins. This family of proteins contribute between 300 and 400 copies of the postsynaptic density protein 95 (PSD-95) to the PSD. Slightly lower numbers for other members of the family (PSD-93, SAP 97, and SAP102) are found in the PSD (Sheng and Hoogenraad, 2007). In addition, the MAGUKs contain several binding sites, most commonly PDZ binding domains, that are positioned very close to the plasma membrane where they can readily interact with the PDZ-ligand domains of the TARPS. This translates to over 900 binding sites at a given synapse containing no more than one or two hundred total surface-expressed transmembrane proteins of a variety of sorts (**Figure 2**) (Shinohara and Hirase, 2009; Tarusawa et al., 2009). This suggests that the number of scaffolding proteins exceeds the amount of surface expressed AMPARs at the PSD. This potential imbalance could be significant, as excess binding sites have been shown to slow down the diffusion of surface AMPARs in cultured neurons (Czöndör et al., 2012).

Another factor regulating diffusion of surface AMPARs is the density of surface-expressed proteins at the PSD, as seen in multiple freeze-fracture EM images (Shinohara and Hirase, 2009; Tarusawa et al., 2009; Budisantoso et al., 2012; Fukazawa and Shigemoto, 2012; Holderith et al., 2012; Choquet and Triller, 2013). **Figure 2** shows examples of those images for a very small synapse (top row) and a large synapse (lower row). The large synapse shows that about 50% of the postsynaptic membrane is filled with small membrane-bound particles (quantified here by Image J). There are over 250 particles in this spine, including 79 particles labeled with an anti-GluR1 (now GluA1) antibody and about 30 labeled by an anti-NR2B (now GluN2B) antibody (**Figure 2**, lower row). The rest of these membrane-bound particles may belong to either mGluR1 and 5, Ca^2+^ and K^+^ channels, neuromodulatory receptors, transsynaptic proteins, or other ionotropic channels (Sheng and Hoogenraad, 2007).

This large amount of membrane proteins coupled with the abundance of scaffolding proteins in the PSD raises a question: To what extent does postsynaptic packing density, including excess MAGUKs or physical membrane proteins, impact intrasynaptic receptor diffusion? Li and Blanpied (2016) addressed the effect of MAGUK binding on protein diffusion rates at the PSD. They engineered a construct containing a pH-sensitive form of GFP (super ecliptic phluorin, or SEP) fused to the transmembrane (TM) domain of the C-terminus tail of stargazin, a protein that binds to AMPAR. The C-terminus targets stargazin to the PSD proteins (PSD-95/SAP-90 and related PDZ proteins). To study mobility, they used single particle tracking of surface-expressed SEP-TM protein and used the 647N ATTO-coupled anti-GFP nanobody to extracellularly label the SEP proteins. These live-labeling experiments showed particles with a range of mobility that was dependent on the location of the particle with respect to the PSD. They found that SEP proteins showed free diffusion at extrasynaptic sites, but highly confined motion within the PSD. Specifically, the mean instantaneous diffusion coefficient (D_inst_), a measure of average mobility, was 0.02 μm^2^/s for extrasynaptic particles, 0.0006 μm^2^/s for synaptic particles, and even slower for particles in zones heavily enriched with PSD-95. This constrained diffusion in the PSD is expected since the PDZ ligand domains of TARPS are responsible for trapping and immobilizing stargazin, and the associated AMPARs, at synapses (Bats et al., 2007).

In addition to the immobilization of surface-expressed AMPARs by binding to the MAGUKs, Li et al. (2016) also evaluated how the presence of physical obstacles, in the form of membrane proteins in the PSD, affect the diffusion of intrasynaptic AMPAR. In contrast to the previously accepted view, Li et al. (2016) found that the PSD most strongly traps larger receptors (i.e., AMPARs and NMDARs) and that this trapping is independent of the receptor’s interaction with scaffolding proteins. In simulation studies, they found that AMPARs were only able to move fast within specific areas of the PSD that had low PSD-95 expression; these zones, termed “conducting paths,” permitted free diffusion of surface proteins such as membrane channels. These conducting paths may correspond to the empty space seen in the freeze fracture replicas in **Figure 2**. In addition, Li et al. (2016) found that the PSD also has patches devoid of PSD-95 within which AMPAR diffusion is corralled. The small range of AMPAR movement happened in small nanodomain-like regions as observed in (Nair et al., 2013; Cai et al., 2014; Constals et al., 2015; Li et al., 2016; Lee et al., 2017). Interestingly, their effects were independent of the size of the postsynaptic PSD tested (0.15 μm^2^ and 0.45 μm^2^). The reason for this is unknown but it may be because the numbers of both AMPARs and scaffold proteins increases linearly with the size of the postsynaptic spine. One caveat is that the smaller spines analyzed in this paper are already large in size in relation to what is considered a small spine (spine sizes < 0.05 μm^2^); thus the effects of molecular crowding may be different for very small stubby spines with relatively low particle density. Taken together, these recent studies have shown that molecular crowding at large spines constrains the speed, range, and directionality of movement for intrasynaptic AMPARs.

### AMPAR Diffusion Coefficient (D_inst_)

Knowing the true distribution of AMPAR mobility is very complex. The actual distribution of D_inst_ for AMPARs depends on the developmental age of the neurons (Czöndör et al., 2012), the imaging technique used, and the size of the fluorescent label marking the AMPARs (Borgdorff and Choquet, 2002; Ehlers et al., 2007; Groc et al., 2007; Heine et al., 2008; Nair et al., 2013; Li et al., 2016; Lee et al., 2017). In general terms, the values of diffusion obtained with the most recently developed imaging techniques show intrasynaptic AMPARs moving very little (Nair et al., 2013; Cai et al., 2014; Lee et al., 2017), whereas the diffusion values obtained using commercially available big quantum dots (bQDs) show the AMPARs moving quite a lot (Heine et al., 2008; Petrini et al., 2009; Opazo et al., 2010; Sainlos et al., 2011). Notably, the values of diffusion obtained with more recent techniques are actually much slower than what is required for the presumed role of AMPAR mobility in paired-pulse stimulation (see mathematical modeling studies, section below). For example, the results in Cai et al. (2014), Li and Blanpied (2016), Li et al. (2016), and Lee et al. (2017) and from Nair et al. (2013) suggest that AMPARs cannot move fast enough to participate in paired-pulse depression. In the original hypothesis of closed receptors exchanging with desensitized ones during paired-pulse stimulation, the receptors need to move with an average D_inst_ of 0.1 μm^2^/s, or at least 70 nm in total. This is so that AMPARs can move from a zone of low glutamate concentration far from the site of release toward the epicenter of glutamate release, which is at least 250 nm in diameter. Receptors that are too close to the zone of initial glutamate release sites will be gated by glutamate and become desensitized. Even if some of the closed AMPARs do move that long distance, the likelihood that the movement will be directed toward the site of release is very low due to the stochastic nature of AMPAR diffusion. More likely than not, the AMPARs will move in the wrong direction.

The low number of fast moving AMPARs suggests that if AMPAR mobility plays a role during paired-pulse depression, some additional mechanism should be at play. Constals et al. (2015) proposed that synaptic glutamate can accelerate the mobility of activated AMPARs. Their idea was that prolonged glutamate stimulation triggers a conformational change that favors the dissociation of the AMPARs from the TARP subunits (Tomita et al., 2004). The authors reasoned that this could allow the AMPARs to move away from the site of glutamate release at a much greater speed. In their experimental setup, they showed that glutamate concentrations high enough to promote gating modestly increased the mobility of AMPARs. Upon bath applications of glutamate, AMPARs go from exploring an area of 0.0025 μm^2^ (or an area corresponding to within a 50 nm circle) to 0.0075 μm^2^ (within a 86.6 nm circle). In these experiments they also observed a slight shift toward greater mobility with a low concentration of glutamate. However, most importantly, during realistic conditions of glutamate release such as following glutamate uncaging, the authors observed very little change in mobility. Under these conditions, no alteration in AMPAR mobility was observed in the first time point of the mean-square displacement (MSD) curve (50 ms time step)^∗^ as seen in Figure 6C of Constals et al. (2015). Moreover, the average D_inst_ after glutamate stimulation was reported to be about 0.01 μm^2^/s, or tenfold lower than the required D_inst_ of 0.1 μm^2^/s. In addition, the non-stimulated spines showed a reduction in mobility. Therefore, it is still unclear how AMPAR/TARP dissociation will impact synaptic transmission during paired-pulse depression.

Taken together, the evidence suggests that the high packing density of the PSD and the large number of surface expressed proteins impose a great restriction to the mobility of synaptic AMPARs. Accordingly, recent data shows that the majority of AMPARs (∼86%) do not move more than 25 nm in 50 ms which supports the view that the PSD is an environment of slow diffusion (Li et al., 2016).

**FIGURE 3:**
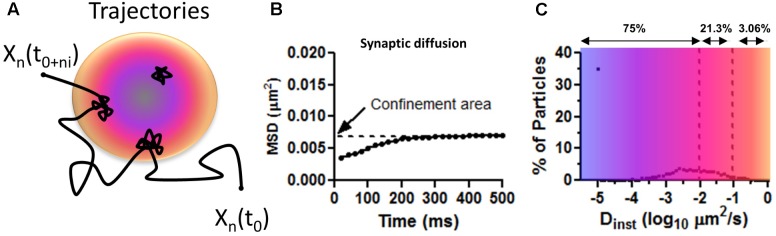
Mean squared displacement calculations on surface AMPAR as approximated values from Figure 1C in Nair et al. (2013). **(A)** Trajectory representing the diffusion of AMPAR on the plasma membrane of neurons. Long steps are seeing at extra PSD areas and confined movements within the PSD (colored disk). Blue is to illustrate a zone of PSD-95 concentration and orange zone with less PSD-95. **(B)** Average distribution of MSD curves as shown by Nair et al. (2013). Notice the low values of displacement for synaptic particles. Confined area (Y intercept) indicates the total area covered (of 0.007 μm^2^ or a radius of 47 nm) for the duration of the imaging (500 ms). **(C)** The average percentage of particles versus the instantaneous diffusion coefficient (D_inst_). Number above shows the total number of particles, as percentage, with diffusion lower than the upper bound. Notice most particle are virtually immobile D_inst_ < 0.01 μm^2^/s showing corralled diffusion.

^∗^These are the values extracted from the MSD, or abscissa, for all two consecutive time points of all particles. The D_inst_ is obtained from the linear fit to the first 3–4 points of the MSD versus time curve for each individually tracked particle (**Figure 3B**). The MSD curve is calculated by squaring the mean distance for the duration of a time step according to the MSD formula:

$$MSD\equiv \left\{{\left(x-x_{0}\right)}^{2}\right\}=\frac{1}{N}\sum_{n=1}^{N}{\left(x_{n}+x_{n}\left(0\right)\right)}^{2}$$

Where N is the number of particles that are averaged, X_n_ (0) is the reference position for each individual particle, X_n_(t) is the particle position determined at that moment in time t. In most experiments, the precision accuracy of drift-corrected recordings is below the total distance traveled by the AMPARs in one time step or 30 nm. Average single time steps or average D_inst_ should accurately describe differences in behavior of these particles.

### Mathematical Models

As stated above, the participation of AMPAR mobility during paired-pulse depression supposedly requires that AMPARs move fast with average D_inst_ of 0.1 μm^2^/s (Heine et al., 2008; Choquet, 2010; Freche et al., 2011; Constals et al., 2015; Compans et al., 2016). Using this value, Heine et al. (2008) then attempted to construct a realistic model of AMPARs present in a PSD, those moving around in the plasma membrane, and those which undergo gating following glutamate binding as described in Raghavachari and Lisman (2004). They showed a good relationship between recovery from paired-pulse depression (pulses 50 ms apart) and the rates of postsynaptic diffusion. When the authors set the average D_inst_ to 0.1 μm^2^/s, which we argue is too high, based on the current results, discussed above, the simulated AMPAR-mediated response showed that postsynaptic surface AMPAR mobility helped recover the synaptic response to about 70% of the first stimulus, thus, experiencing some paired-pulse depression. This is in stark contrast to the results obtained when the average D_inst_ was set to zero to mimic crosslinking between AMPARs. Under these conditions the simulated response was only about 30% of the initial response. However, these modeling results agreed extremely well with experimental data that used primary and secondary antibodies to crosslink AMPARs and thus reduce the mobility of the receptors. Nevertheless, the selected values of mean D_inst_ is too high in comparison to our current understanding. In the next section we will discuss several technical limitations that led the authors to reach their conclusion.

### Technical Limitations

The first two factors that go hand in hand are: (1) the use of an artefactually high value of instantaneous diffusion coefficient of D_inst_ and the (2) the technical limitations of using bQDs to extract the values of D_inst_ from the MSD curve. In the experiments using bQDs, over 60% of particles move with D_inst_ larger than 0.1 μm^2^/s. As a result, many modeling papers have used this value as the mean D_inst_ value (Heine et al., 2008; Tolle and Le Novère, 2010; Czöndör et al., 2012; Nair et al., 2013). However, when smaller fluorescent probes are used, most labeled particles move with D_inst_ lower than 0.1 μm^2^/s. For example, with overexpressed mEOS3.2-tagged GluA1 subunits (mEOS3.2 is essentially a GFP-like probe, and is much smaller than a bQD), about 75% of surface-expressed AMPARs move slower than 0.01 μm^2^/s or move about 22 nm, 96% of surface AMPARs move slower than 0.1 μm^2^/s or move about 50 nm in 50 ms, and only the remaining 4% of surface AMPARs move slower than 0.5 μm^2^/s, or about 158 nm in 50 ms. (In **Figure 3C**, the approximate values of D_inst_ are obtained from Figure 1 in Nair et al., 2013). For endogenous GluA2-containing AMPARs, labeled with an anti-GluA2 antibody coupled to ATTO 647N, corresponding to a “intermediate” size probe but still small compared to a bQD, the distribution of D_inst_ is very similar to the results obtained using the mEOS3.2 tagged GluA1 subunits. The GluA2 labeled with the antibody showed slightly lower values of D_inst_ (i.e., they move slower) than receptors tagged with the mEOS3.2-GluA2 (Nair et al., 2013); although the distributions almost look the same. In general terms, ∼85% of GluA2-containing AMPARs, labeled with the anti-GluA2 antibody, and 75% of mEOS3.2-tagged AMPAR receptors do not move more than 50 nm in 50 ms (Nair et al., 2013; Cai et al., 2014; Li and Blanpied, 2016; Li et al., 2016; Lee et al., 2017). Therefore, these findings suggests that the majority of synaptic localized AMPAR don’t move with average D_inst_ of 0.1, but move much slowly than previously thought.

We argue that smaller probes give slower values of D_inst_, which off-hand, might seem counter-intuitive. The reason is that with smaller probes, the AMPAR are more likely to be labeled inside of the synapse, where AMPAR movements are more constrained. For example, Lee and colleagues noticed that when AMPARs are labeled with fluorophores of smaller sizes, they are primarily in the synapse—75% of AMPARs are synaptic when labeled with small quantum dots (sQDs) and 90% of AMPARs are synaptic when labeled with the small CF633- or Atto647N-tagged streptavidin. However, only 5% of AMPAR is synaptic when labeled with big QDs. As seen by others (Heine et al., 2008; Petrini et al., 2009; Opazo et al., 2010; Sainlos et al., 2011), the labeling with bQDs in Lee et al. (2017) mostly happens at extrasynaptic sites and gives very high values of D_inst_ (Howarth et al., 2008; Lee et al., 2017); suggesting that the results obtained using bQDs misrepresent the true localization of surface AMPARs and values of D_inst_. In support of this idea, Lee and colleagues rarely or never observed the big QDs re-entering the postsynaptic spines in over 1 h of imaging; supporting the idea (to be discussed next) that the bQDs have a hard time accessing the synaptic cleft. However, when Lee and colleagues imaged neurons labeled with small QDs, the small QDs remained inside the postsynaptic spines for at least 15 min; supporting the idea that small QDs have unobstructed access to the synaptic cleft and synaptic AMPARs have an extremely long residency time.

The exact mechanism of why D_inst_ decreases with a decrease in size of the label is unknown, but the high value of D_inst_ observed with the big QDs could be due to a number of factors: (1) the bQDs might dislodge the AMPARs from the PSD, (2) bQDs may not be able to penetrate the synaptic cleft well enough to become anchored by the scaffolding proteins, or (3) bQDs may dissociate the AMPARs from the auxiliary TARPS. The smaller fluorescent probes do not appear to have this problem, probably because they can easily penetrate the synaptic cleft and are less likely to sterically interfere with the stability of the synaptic AMPARs. More reassuring is the fact that all values of D_inst_ obtained using the smaller labels—whether the small QDs (<12 nm diameter) or the streptavidin-organic fluorophores (<5 nm diameter)—are similar. Thus, we believe that the values of D_inst_ obtained using big commercial QDs are artefactual in nature while the values obtained using the small labeling tags most closely track the true mobility of endogenous synaptic AMPARs (Nair et al., 2013; Cai et al., 2014; Li and Blanpied, 2016; Li et al., 2016; Lee et al., 2017). Therefore, the mathematical models implemented using the potentially artefactual high values of D_inst_ may need to be re-evaluated using more realistic distributions of D_inst_.

An additional factor is the application of antibodies to live cells. This is one of the most commonly used techniques in biological research. In most of the studies using antibodies to perform live labeling of surface AMPARs, the neurons are labeled while maintained at 4°C. This is done to slow the rates of endocytosis and to allow sufficient time to label all surface-expressed receptors. In contrast, in the experiments where AMPARs were crosslinked together, Heine et al. (2008) incubated the neurons with antibodies at physiological temperatures (i.e., 37°C) for long periods (longer than 30 min). This is the time that it takes to do the primary incubation (10 min), washes (2 min), secondary incubation (10 min), washes (2 min), mounting the sample in the imaging chamber (2–3 min), positioning the sample in the microscope stage (1–2 min), finding the neurons (5–15 min), preparing pipettes for whole cell paired recordings (10–15 min), and waiting for the whole cell patch to equilibrate with intracellular solution (2–5 min).

The physiological consequences of long incubations of neurons with antibodies against surface AMPARs at physiological temperatures have been studied in a recent paper by Peng et al. (2015). In this work, the authors applied cerebrospinal fluid (CSF) from patients with limbic encephalitis to living cultured neurons. This CSF contained IgGs that recognized the extracellular N-terminus domain of the AMPARs. One hour after incubating with the CSF, they noticed a reduction in the number of surface AMPARs and a concomitant increase in the amount of intracellular AMPARs present in the lysosome (Peng et al., 2015). This same effect was also triggered if the surface AMPARs were crosslinked with commercial antibodies against the GluA1 or GluA2 subunit of the AMPARs. More importantly, the encephalitis patients’ CSF reduced the amplitude and frequency of miniature EPSCs (mEPSCs), an effect that became evident 1 h after antibody application. Interestingly, this effect is not isolated to AMPARs, as antibodies against NMDARs (Dalmau et al., 2011), potassium channels (Sun and Li, 2013; Sun et al., 2016), FGFR1 (Opaliński et al., 2017), ErbB3 (Belleudi et al., 2012), Human Respiratory Syncytial Virus Fusion Protein (Leemans et al., 2017), and acetylcholine receptors (Lee et al., 2014) also trigger a reduction of their respective target proteins. These studies suggest that antibodies against surface proteins induce internalization after the proteins are crosslinked if the samples are not maintained in the cold. Maintaining labeling at ∼4°C is apparently essential.

Another issue related to the use of antibodies to crosslink surface AMPARs is the penetration of the antibody complexes (used as the crosslinking agent) in the synaptic cleft. (Here we use the term crosslinking complex to refer to the macromolecular assembly composed of AMPARs, connected to other AMPARs via primaries and secondary antibodies.) The minimal unit of a crosslinking complex is achieved when at least two AMPARs are recognized by either one primary antibody or by two primary antibodies tethered together by a secondary antibody. The number of antibodies present in the crosslinking complex depends on: (1) the abundance of epitopes, (2) the access of the antibody to the AMPAR, and (3) on how much primary and secondary antibodies are applied to the sample. If high concentrations of primary antibodies are applied to saturate all epitopes, then more than two AMPARs are expected to be crosslinked. In a hypothetical scenario that three AMPARs are crosslinked, this crosslinked complex will contain 3 AMPARs, between 2 and 4 primary antibodies, and between 2 and 4 secondary antibodies. In terms of size limitations, the linear size of the crosslinking complex is at least over 24 nm is size when bound to the N-terminus domain of the AMPAR. In terms of the width of these complexes, the crosslinking antibodies could form a large macromolecular complex that may surpass several million Dalton in size and exceed the size of the big QDs in hydrodynamic diameter. (see Supplementary Figure 8 in Heine et al., 2008, where this large size is very evident). Thus, the crosslinking complex will form a large extracellular molecular complex that may have limited penetration to the crowded space of the synaptic cleft.

These reasons support the idea that in live-labeling crosslinking experiments, the AMPAR crosslinking complex may have limited penetration to the synaptic cleft. The issues related to impaired penetration to the synaptic cleft by large tags (i.e., larger than a primary antibody or big QDs) have been studied in Howarth and Ting (2008), Nair et al. (2013), Chamma et al. (2016), and Lee et al. (2017). In these studies they show that small size probes penetrate the synaptic cleft, while the larger tags (i.e., primary antibodies and big QDs) rarely produce any synaptic staining in live cells. For example, Nair et al. (2013) compared the diffusion coefficients of both the mEOS3.2-tagged GluA2-containing AMPARs and the endogenous GluA2-containing AMPARs labeled using the ATTO 647N-labeled anti-GluA2 primary antibody. In both cases, the authors showed good synaptic labeling, as expected via the arguments stated here (Nair et al., 2013). Furthermore, the authors confirmed the results from the super-resolution imaging with EM. Overall, the study of Nair and colleagues show that labeling of synaptic AMPARs can be achieved with slightly large probes, such as antibodies against the GluA2 subunits. However, the live imaging using the mEOS3.2 probe provided better coverage of the synaptic structure than the labeling achieved with the anti-GluA2 antibody. This implies that smaller tags provide superior synaptic labeling.

In Chamma’s study, they specifically addressed the effect of different-sized tags on the ability to provide good synaptic labeling of neuroligin in live labeling experiments on cultured neurons. In general terms they observed that smaller-sized tags yielded better synaptic labeling than larger size tags. The authors scored localization of the labeled neuroligin in relation to the center of the Homer labeling (used as a marker of the PSD) as a proxy for the penetration of the extracellularly applied labeling molecule. Good penetration was scored as perfect axial alignment with the Homer labeling; partial penetration was scored as partial overlay. They used labels of different sizes: the small monomeric streptavidin (mSA 12.5 KDa or less than 4 nm in size), tetrameric streptavidin (SA 52.8 KDa and slightly over 5 nm in size), and a full-sized primary antibody against neuroligin (150 kDa, over 12 nm in size) all labeled with Alexa647. They found that the full-sized antibodies and tetrameric SA stained neuroligin outside of the Homer label, while the small monomeric streptavidin stained neuroligin right in the center of the Homer label. This suggests that sometimes even the primary antibodies have a limited penetration to the synaptic cleft (see Figures 1, 2 of Chamma et al., 2016).

This effect is not just specific to neuroligin, as Lee et al. (2017) have replicated this result using mSA, SA and a very small nanobody (against GFP-AMPAR) while studying the dynamics of AMPARs in the same experimental system. In this paper, Lee et al. (2017) studied how the quality of synaptic labeling and the mobility of surface AMPARs is affected by the size of the fluorophore, tag, and a combination of the two. In general terms, the Selvin group found that large fluorescent probes, which increase the size of the complex, produce very little synaptic labeling. As discussed previously, Lee and colleagues used three labels: commercial big QDs (∼25 nm in hydrodynamic diameter), small homemade QDs (∼10–12 nm in hydrodynamic diameter), and the small monomeric streptavidin probes (∼4 nm in size). They found that over 90–95% of bQD-labeled AMPARs were found at extrasynaptic membranes or dendrites, while under the same conditions over 90% of AMPARs labeled with either small QDs or small monomeric streptavidin probes were found associated with the PSD. The data of Lee and colleagues suggests that the crosslinking complex when bound to the GluA subunits will surpass the size of the small molecules that can penetrate the synaptic cleft in live labeling experiments. This further suggests that primary antibodies and to a greater extent crosslinking complexes have limited access to the synaptic cleft. In addition, single primary antibodies and crosslinking complexes may trigger the loss in some unknown fashion of surface expressed receptors. Thus, it may be difficult to dissociate the effects of AMPAR diffusion from crosslinking-induced redistribution of AMPARs in synaptic transmission.

## Alternative View and Future Directions

The various technical issues mentioned above suggest that alternative ideas are needed to help explain the proposed role of postsynaptic AMPAR mobility in paired-pulse depression. As discussed above, the link between postsynaptic AMPAR mobility and paired-pulse depression was forged from antibody crosslinking experiments that led Heine and colleagues to the conclusion that crosslinking AMPARs decreases their mobility and increases the degree of paired-pulse depression. A link between paired-pulse depression, mobility, and AMPAR desensitization was established by the drug cyclothiazide, which eliminates desensitization of AMPARs (Trussell et al., 1993). Because the decrease in paired-pulse depression is rescued by cyclothiazide, the conclusion was that desensitization of postsynaptic AMPARs is responsible for the observed increase in paired-pulse depression. Furthermore, the authors concluded that the desensitized AMPARs are replaced by the non-desensitized AMPARs during paired-pulse depression. Given the previously discussed issues with these experiments, how is it that crosslinking and cyclothiazide are affecting the recovery from paired-pulse depression?

**FIGURE 4:**
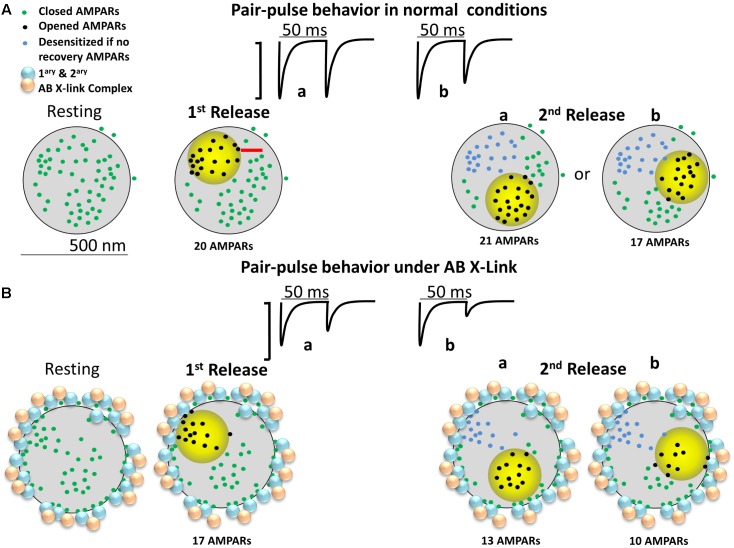
Diagram of a large mushroom spine with realistic AMPAR content randomly distributed thought the space as shown by Shinohara and Hirase (2009). Zone of high glutamate concentration is illustrated by the yellow circle. Three different states for AMPAR are assigned: closed (green color), opened (black), and desensitized (blue). AB X-ling complex is depicted by solid gold and blue particles. **(A)** Control synapse. Three states are shown: resting (left), 1st pulse (middle left), and two possible sites for the 2nd release (right a and b). Response for paired-pulses is shown above. Estimated synaptic response is shown above as a one to one correspondence between number of AMPAR and size of response. **(B)** The cross-linking synapse. The AB particles decorate the perisynaptic area. AB complexes induced redistribution of AMPARs is shown.

**Figure 4** is an attempt to develop an alternative conceptual framework to: (1) explain how antibody crosslinking may induce an effect on AMPAR mobility, (2) explain how a drug that blocks desensitization may seem to rescue the enhancement in paired-pulse depression, and (3) contrast the conceptual ideas (i.e., presence of nanodomains, alternating nano-zones of glutamate release, intrasynaptic mobility, large PSDs, etc.) that are discussed in Figure 1 of Compans et al. (2016) and Figure 2 of Choquet and Triller (2013). We show a top–down post-synapse under resting conditions (**Figure 4A**) and what we believe would happen to the AMPARs after crosslinking (**Figure 4B**). We present a synapse with semi-realistic dimensions and appropriate AMPAR spacing. The AMPARs are randomly distributed throughout the synapse and some are clustered in nanodomains as shown in **Figure 1**. The activation zone of AMPARs is 250 nm in size (Raghavachari and Lisman, 2004) and is represented by the yellow circle. This is the area in which glutamate release from the pre-synaptic membrane is highest and follows the original model proposed by Heine et al. (2008). The AMPARs in the yellow zone are in three different states: closed, open, and desensitized. The first and second pulses represent “glutamate” release in the same fashion of paired-pulse stimulation as in the model of Compans et al. (2016). AMPARs enter the desensitized state quickly, though the rate of exit from desensitization is also rather fast, with a half-time recovery of 26 ms (Robert and Howe, 2003). Because not all AMPARs will be exposed to high glutamate concentrations, a fraction will remain non-activated/non-desensitized. As discussed before, we do not consider intrasynaptic diffusion because in 50 ms the majority of AMPARs will have moved less than 40 nm. Therefore, we only describe hypothetical cases where the first and second vesicles activate different nanodomains as seen in Tang et al. (2016). This is referred to as “random release” (Compans et al., 2016). For simplicity of discussion, there are at least three independent nanodomains with enough AMPARs to elicit a synaptic response. We predict that these synapses will follow fast synaptic transmission only if glutamate release occurs at distinct zones of the post-synapse. In the control condition, the paired-pulse stimulation shows that the first glutamate pulse (left) could activate about 20 AMPARs and the second pulse about 21 AMPARs or 17 AMPARs (**Figure 4**). One scenario is expected to produce either little (**a** of 2nd release) or more noticeable paired-pulse depression (**b** of 2nd release) simply because a different number of AMPARs could be activated.

In **Figure 4B**, the crosslinking condition is represented by primary and secondary particles, or equivalently, large quantum dots, present at the perisynaptic area. The preferential positioning of the antibody complex at perisynaptic zones is based on the papers showing that large macromolecules or particles enter the center of the synaptic cleft of excitatory synapses with greatly decreased frequency (5–10%) (Howarth et al., 2008; Chamma et al., 2016; Lee et al., 2017). The depicted localization assumes that the antibody complex, given enough time, induces redistribution of synaptic AMPARs to the perisynaptic area before the complex is internalized. This logic follows the effects of anti-GluAs human encephalitis antibodies which trigger a time-dependent reduction in mEPSC amplitude and frequency (Peng et al., 2015). We do not expect that in short time domains (tens of minutes) all the AMPARs will be redistributed; just some AMPARs will be uncoupled from the center of the synaptic cleft.

Under these conditions, we predict that a simple antibody-induced redistribution will lead to strong alterations in synaptic transmission. One plausible scenario is that under the crosslinking condition, the first glutamate pulse will activate a lower fraction of AMPARs (about 17) and the second pulse about 13 AMPARs (**a** of 2nd release, lower row) or 10 AMPARs (**b** of 2nd release, lower row). This lower number is the product of the AMPARs that were closer to the perisynaptic area getting redistributed to the crosslinking complex. This scenario predicts a decrease in the number of activated AMPARs to the second glutamate pulse and an increase in paired-pulse depression that is independent on AMPAR desensitization. The major determining factor in this scenario is the remaining AMPARs present in the center of the synaptic cleft after the crosslinking manipulation.

So how could cyclothiazide rescue this particular “phenomenon” and help explain the physiological results of Heine et al. (2008)? Besides its effect on AMPAR desensitization, cyclothiazide potentiates AMPAR currents and increases glutamate affinity (Fucile et al., 2006). In the crosslinked synapse, the presence of cyclothiazide could potentiate the 13 or 10 AMPARs within the 250 nm cloud of glutamate. Furthermore, it could promote the gating of AMPARs that are close to the 250 nm cloud because these receptors are now within the activation range of glutamate because cyclothiazide increases their affinity toward glutamate and decreases their desensitization (Robert and Howe, 2003). This could activate between 5 to 10 extra AMPARs which are positioned in the zone where glutamate concentration is in the low micromolar range. These are the green dots almost touching the yellow circle and are positioned beyond 125 nm from the site of vesicular release. These two effects of cyclothiazide, potentiation and increased glutamate affinity, can fully rescue the effects of crosslinking in a fashion independent of its effect on AMPAR desensitization.

This conceptual model provides an alternative view on the effects of crosslinking on fast synaptic transmission and stresses the importance of better controls on these types of experiments. Therefore, new ideas and better methods (genetic and fluorescent probes) should be proposed to disentangle the effects of antibody-induced redistribution of AMPARs from the mobility of AMPARs in order to understand the true role of mobility in fast excitatory synaptic transmission. If our ideas are correct they should be testable with available technologies. One such way would be to implement the antibody-induced crosslinking approach to immobilize postsynaptic AMPARs and test the effects of the crosslinking on paired-pulse depression as a function of time. We predict that the effects of the antibody crosslinking approach would have a time-dependent effect, with the crosslinking antibody having weak effects on electrophysiological recordings made shortly after the end of the crosslinking approach (∼30 min) and stronger effects after a longer period has elapse (>1–2 h after crosslinking). These measurements should be complemented with superresolution microscopy (dSTORM on fixed neurons) to colocalize the crosslinked AMPAR complex in relation to the postsynaptic density marker. If we are correct, we predict that the localization experiments may show a time-dependent redistribution of the antibody complex away from the PSD-95-positive cluster and an increase in the staining at extrasynaptic membranes. There may even be an increase in the colocalization of these clusters with the endocytic pits (Petrini et al., 2009).

All of this evidence suggests that better tools are needed that can reduce the mobility of AMPARs without interfering with their distribution or biophysical properties. A plausible way would be to generate knock-in mice where the PDZ binding domain of TARPs is introduced into the GluA1 or GluA2 subunits of the AMPARs. This type of approach was developed in Constals et al. (2015) where a stargazin tandem fusion protein with GluA1 or GluA2 was created. However, the GluA1i tandem construct shows increased steady-state current and time constant and accelerated recovery from desensitization (Morimoto-Tomita et al., 2009). Therefore, alternative approaches need to be implemented. Several other approaches should be carried on to promote protein-protein interaction that come together upon the application of a drug, a light, or a crosslinking protein. Two systems could be implemented that may serve this purpose.

First, a potential starting strategy could be the use of the rapamycin-induced oligomer formation system composed of the FKBP/FRB protein domains (Derose et al., 2013) where the FKBP binding domain is fused to the GluA1 subunit and the FRB is fused to the GluA2 subunit. This system is very reliable in many experimental setups.

A second strategy would be to use two of the four known light-inducible dimerization systems described in the literature. The LOV-Jα and the Phy/Pif systems offers the greatest advantage because they have very fast on/off kinetics (Karunarathne et al., 2015). These two light-induced protein-protein interaction systems offer the distinct benefit of being reversible. The LOV-Jα system shows a slower relaxation time while the reversibility of the Phy/Pif system is very fast and is controlled by far-red light. Two major disadvantages of the Phy/Pif system are the need of a cofactor and the large size of the binding domains. The large size of the protein domains in the FKBP/FRB and the light-induced systems may make them hard to implement with the GluA subunits because it could interfere with the folding/assembly of these subunits. A different system that has been successfully implemented for the crosslinking of surface AMPARs consists of engineering an acceptor peptide at the N-terminus domain of the GluA1 subunit and the expression of the ER-targeted biotin ligase (BirA) (Howarth et al., 2005; Penn et al., 2017). Using this system, several groups have labeled surface-expressed GluAs with small quantum dots and fluorescently labeled streptavidin (Howarth and Ting, 2008; Lee et al., 2017). This system has also been used to immobilize AMPARs in both cultured neurons and in organotypic slice preparations (Penn et al., 2017). Penn et al. (2017) tested the role of AMPAR mobility in the expression of LTP and observed that the biotinylation manipulation completely blocked the expression of pairing-induced LTP. This form of LTP is thought to require the release of new AMPARs to the plasma membrane and which are then enriched at the synapse via lateral mobility (Citri and Malenka, 2008; Makino and Malinow, 2009). The authors concluded that with LTP, the AMPARs already present at the plasma membrane are then recruited to the potentiated synapse. However, an equally plausible hypothesis is that small intrasynaptic mobility that rearranges synaptic AMPARs is required for the expression of LTP. The small intrasynaptic mobility may be required for the increase in density of AMPARs that become concentrated at the transsynaptic nanocolumns following LTP as has been observed for PSD-95 (Tang et al., 2016). The precise mechanism of how LTP recruits AMPARs via lateral mobility awaits further research. Unfortunately, while streptavidin virtually blocked the mobility of surface AMPARs and consequently the expression of LTP, it had virtually no effect on the response of the excitatory synapses to paired-pulse stimulation. Unlike the initial proposition by the authors that AMPAR mobility plays a role in paired-pulse stimulation, the role of AMPAR mobility appears to be a phenomenon mostly required for the expression of LTP and not for paired-pulse stimulation. These results are in complete agreement with our interpretation that AMPAR mobility in synapses in culture, organotypic slices, or *in vivo* is too slow to participate during periods of repetitive presynaptic stimulation.

The experiments mentioned above should provide new exciting ideas about the function of canonical excitatory synapses. In conclusion, the exciting idea that AMPAR mobility plays a role in tuning fast synaptic transmission deserves further studies.

## Author Contributions

JD and PS evaluate all stages in the preparation of the manuscript.

## Conflict of Interest Statement

The authors declare that the research was conducted in the absence of any commercial or financial relationships that could be construed as a potential conflict of interest.
